# (−)-Methyl-Oleocanthal, a New Oleocanthal Metabolite Reduces LPS-Induced Inflammatory and Oxidative Response: Molecular Signaling Pathways and Histones Epigenetic Modulation

**DOI:** 10.3390/antiox11010056

**Published:** 2021-12-27

**Authors:** Tatiana Montoya, Catalina Alarcón-de-la-Lastra, María Luisa Castejón, Juan Ortega-Vidal, Joaquín Altarejos, Marina Sánchez-Hidalgo

**Affiliations:** 1 Department of Pharmacology, Faculty of Pharmacy, Universidad de Sevilla, 41012 Sevilla, Spain; tmontoya@us.es (T.M.); calarcon@us.es (C.A.-d.-l.-L.); mcastejon1@us.es (M.L.C.); 2 Department of Inorganic and Organic Chemistry, Faculty of Experimental Sciences, Campus de Excelencia Internacional Agroalimentario (ceiA3), University of Jaén, 23071 Jaén, Spain; jov00007@red.ujaen.es (J.O.-V.); jaltare@ujaen.es (J.A.)

**Keywords:** antioxidant, histones, inflammation, macrophages, metabolite, methylation, oleocanthal, olive oil

## Abstract

The antioxidant and anti-inflammatory responses of (−)-methyl-oleocanthal (met-OLE), a new metabolite of the extra virgin olive oil (EVOO) phenolic oleocanthal (OLE), were explored in lipopolysaccharide (LPS)-induced murine peritoneal macrophages. Possible signaling pathways and epigenetic modulation of histones were studied. Met-OLE inhibited LPS-induced intracellular reactive oxygen species (ROS) and nitrite (NO) production and decreased the overexpression of the pro-inflammatory enzymes COX-2, mPGES-1 and iNOS in murine macrophages. In addition, met-OLE was able to significantly decrease the activation of p38, JNK, and ERK mitogen-activated protein kinases (MAPKs) and blocked canonical and non-canonical inflammasome signaling pathways. On the contrary, met-OLE upregulated haem oxigenase 1 (HO-1) and nuclear factor (erythroid-derived 2)-like 2 (Nrf-2) expression in treated cells. Finally, met-OLE pretreated spleen cells counteracted LPS induction, preventing H3K18 acetylation or H3K9 and H3K27 demethylation. Overall, these results provide novel mechanistic insights into the beneficial effects of met-OLE regarding the regulation of the immune–inflammatory response through epigenetic changes in histone markers. This revealing evidence suggests that the methylated metabolite of OLE may contribute significantly to the beneficial effects that are associated with the secoiridoid-related compound and the usual consumption of EVOO.

## 1. Introduction

Macrophages are major components of the innate immune system and play a critical role in modulating inflammatory and immune responses [1]. Extracellular bacterial lipopolysaccharide (LPS) acts as pathogen-associated molecular pattern and is recognized by the Toll-like receptor (TLR)-4, inducing macrophages to an activated state, producing pro-inflammatory cytokines and chemokines and enhancing the expression of inflammatory-related enzymes, such as inducible nitric oxide synthase (iNOS), cyclooxygenase (COX)-2 and microsomal prostaglandin E synthase (mPGES)-1, which synthesize nitric oxide (NO) and prostaglandin (PG)E_2_, respectively [2]. Additionally, LPS-stimulated macrophages disrupt the balance of the intracellular reduction–oxidation state, leading to oxidative stress, usually accompanied by damage that is mediated by reactive oxygen species (ROS) [3]. The process of gene expression of these pro-inflammatory mediators involves multiple signal transduction pathways, which are mainly through mitogen-activated protein kinases (MAPKs), nuclear transcription factor-kappa B (NF-κB), janus kinase/signal transducer and transcription activator of transcription (JAK/STAT) or inflammasome activation. Furthermore, the nuclear factor (erythroid-derived 2)-like 2 (Nrf-2)/haem oxygenase-1 (HO-1) antioxidative axis, which exerts a regulative function on the activation of ROS, MAPKs, and inflammasome signaling pathways, is repressed in the event of the induction of the activated macrophages state [2,3].

Emerging evidence suggests that epigenetic processes that affect gene expression without causing changes in the nucleotide sequence occur after external stimuli exposure, and may contribute to the pathophysiology of inflammatory processes [4]. In particular, histone H3 methylation at lysine 9 (H3K9), one of the most conserved epigenetic markers, is correlated with gene silencing and the modulation of immune cell differentiation and immune responses, and therefore, influences the outcome of inflammation. Similarly, H3 acetylation on lysine 18 (H3K18ac) is a permissive marker on genes encoding cytokines that correlate to inflammation, such as interleukin (IL)-1β, IL-6 or IL-17 [5,6,7]. Furthermore, post-translational histone modifications have emerged as prospective therapeutic targets. Understanding the cross-link of these mechanisms could be crucial in designing new immune–inflammatory approaches that are effectively related to several inflammatory diseases [8].

In this context, dietary nutrients could modify physiological and pathological processes through critical epigenetic mechanisms, promoting modifications of gene expression without alteration of the genetic code. In particular, specific, functional foods such as extra virgin olive oil (EVOO) have displayed anti-inflammatory activities in human macrophages through epigenetic mechanisms [9].

The health-promoting properties of EVOO have been correlated with its peculiar chemical composition. EVOO polyphenols are minor secondary metabolites that have been widely studied due to their wide functional versatility, including their anti-inflammatory, antioxidant, cardioprotective, chemopreventive, and neuroprotective properties [10]. Essentially, the anti-inflammatory activities of olive oil, especially phenolic compounds, have recently been linked to their potential to induce epigenetic modifications such as gene expression, DNA methylation and histone modification [10,11].

Examples of the main olive polyphenols are tyrosol, hydroxytyrosol, oleocanthal (OLE), oleacein, olive ligustroside and oleuropein. OLE represents up to 10% of the total polyphenol content in EVOO (0.2–498 mg/kg) [12] and has received more scientific attention, due to its interesting biological activities, both within in vitro and in vivo systems, including anti-inflammatory, antioxidant, cardioprotective, chemopreventive and neuroprotective properties [10,13]. In fact, recently, we have reported the preventive role of dietary OLE-supplemented effects in a collagen-induced arthritis (CIA) murine model and the ability of OLE to diminish the acute inflammatory response in LPS-induced murine peritoneal macrophages [14,15].

Metabolic transformations or the presence of metabolites can affect the pharmacological activity of the pattern compounds. It has been reported that OLE can remain intact in the stomach for up to 4 h and enter the small intestine unhydrolyzed. Then, non-hydrolyzed OLE follows further metabolic reactions related to phase I and II in the liver, namely, hydroxylation or hydration and methylation, respectively. Consequently, López-Yerena et al. (2021) proposed two plausible metabolites of OLE, however, met-OLE was the main circulating conjugate of OLE detected in all tissues analyzed from rats after the acute intake of a refined olive oil containing 0.3 mg/mL of OLE [16]. In relation to biotransformation, the methylated metabolites are mainly metabolized by glucuronidation and sulphation, as they are not substrates for methyltransferases.

Perhaps, the major disadvantage of the EVOO phenols is their temperature instability, photolability, and inadequate pharmacokinetic profile. To reduce these handicaps, methylation of the phenolic hydroxyl groups (O-methylation) may increase the chemical stability of their structures, while conferring greater lipophilicity, increasing their metabolic stability and membrane transport and facilitating absorption and greater oral bioavailability. Recent studies also indicate that the methylation process increases biological activity without altering therapeutic indices [17].

Taking this background into account, the aim of the present work was to investigate the potential antioxidant and anti-inflammatory effects of a new OLE metabolite, (−)-methyl-oleocanthal (met-OLE), in LPS-induced murine peritoneal macrophages. Specifically, intracellular ROS, NO, pro-inflammatory cytokines production (IL-1β, IL-6, IL-17, IL-18, tumor necrosis factor (TNF)-α and interferon (IFN)-γ), and the protein expressions of pro-inflammatory enzymes (COX-2, iNOS, and mPGES-1) were evaluated. In addition, the possible molecular signaling pathways involved in their beneficial effects, such as Nrf-2/HO-1, MAPKs, and the canonical and noncanonical inflammasome, were also studied. Finally, to study the role of the epigenetic mechanisms underlying met-OLE anti-inflammatory effects, we explored met-OLE induced epigenetics changes in histone markers (H3K9me3, H3K27me3 and H3K18ac) and cytokines-correlated production compared to OLE in spleen cells after LPS induction.

## 2. Materials and Methods

### 2.1. Reagents

Solvents used for extraction, analytical thin-layer chromatography (TLC), column chromatography (CC) and fast centrifugal partition chromatography (FCPC), such as diethyl ether (Et_2_O), ethyl acetate (EtOAc), *n*-hexane (Hex), dichloromethane (DCM) and ethanol (EtOH) were of analytical grade and were purchased from VWR Chemicals (Prolabo^®^, Fontenay-sous-Bois, France). Water (H_2_O), used for chromatographic separations, was of ultrapure grade and was produced by Milli-Q water (1.8 MΩ) equipment (Merck^®^, KGaA, Darmstadt, Germany). Acetonitrile (ACN) and methanol (MeOH), used for high-performance liquid chromatography (HPLC), and chloroform, used to determine optical rotation values, were of HPLC grade and were purchased from VWR^®^ (Madrid, Spain). Deuterated chloroform (CDCl_3_) was used to prepare solutions of isolated compounds for nuclear magnetic resonance (NMR) analysis and were purchased from VWR^®^ (Madrid, Spain). Acetic acid (AcOH), used for HPLC, was purchased from VWR^®^ (Madrid, Spain). Silica gel 60 F240 precoated aluminum sheets were purchased from Merck^®^ (Darmstadt, Germany). *N*-methylurea, used to prepare *N*-methyl-*N*-nitrosourea (see Supplementary Material), was purchased from Sigma-Aldrich^®^ (Madrid, Spain). Sodium hydroxide was purchased from VWR Chemicals^®^ (Prolabo, Fontenay-sous-Bois, France).

### 2.2. Instruments

HPLC analyses were performed on a Waters HPLC instrument, equipped with a C18 reversed-phase Spherisorb ODS-2 column, 250 × 3 mm i.d, 5 μm (Waters Chromatogra-phy Division^®^, Mildford, MA, USA), and a photodiode array detector.

Reactions under microwave irradiation were achieved on a CEM Discover monomodal microwave reactor with temperature and pressure internal probes. A sealed vessel was used to perform the reaction.

Purification of synthesized compounds was carried out on a FCPC-200^®^ instrument (Kromaton Technologies^®^, Angers, France) that was fitted with a rotor and with atotal column capacity of 200 mL. Rotation could be adjusted from 0 to 2000 rpm. Solvent was pumped by an Alltech 627 isocratic pump (Alltech Associates^®^, Deerfield, IL, USA). The sample was injected into the FCPC column with a 3725i-038 manual injector (Rheodyne^®^, Cotati, CA, USA) equipped with a 10 mL sample loop. The instrument was equipped with a UV-Vis Linear UVIS 200 detector (Linear Instrument Co^®^, Reno, NV, USA) set at 280 nm.

Mass spectra (ESIMS) were recorded on an Esquire 6000 ion mass spectrometer (Bruker Daltonics, Bremen, Germany) fitted with an electrospray ionization (ESI) interface, operating in positive mode. High-resolution mass spectra (HRMS) were performed on an Agilent 6520B spectrometer (Agilent technologies, Waldbronn, Germany) fitted with a quadrupole time-of-flight (Q-TOF) mass spectrometer.

Proton nuclear magnetic resonance (^1^H NMR) and carbon nuclear magnetic resonance (^13^C NMR) spectra of the isolated and synthesized compounds were recorded on a Bruker Avance DPX 400 spectrometer (Bruker Daltonic GmbH^®^, Rheinstetten, Germany) at 400 and 100 MHz, respectively. Deuterated chloroform, with tetramethylsilane (TMS) as internal reference, was used to dissolve the samples. Coupling constants (*J*) are provided in hertz (Hz) and the multiplicities of signals are reported using the following abbreviations: singlet (s), broad singlet (br s), doublet (d), doublet of doublets (dd), doublet of doublet of doublets (ddd), triplet (t), doublet of triplet (dt), quadruplet (q) and multiplet (m).

Specific rotations ([α]_D_) of chiral compounds were calculated by measuring the corresponding optical rotation (α) in chloroform on a Jasco P-200 automatic polarimeter (Jasco Analytical Instrument^®^, Easton, MD, USA) using cells of quartz with a path length of 1 dm.

### 2.3. Isolation of (−)-Oleocanthal (OLE) from Olive Oil

A sample of OLE was purified from olive oil phenolic extract by a combination of two techniques (fast centrifugal partition chromatography (FCPC) and semi-preparative high-performance liquid chromatography (HPLC)), following the procedure described in Diez-Bello et al., (2019) [18]. In short, an olive oil sample (300 g) was extracted with a mixture of MeOH/H_2_O 8:2, according to a normalized procedure (IOC, 2017), to afford a phenolic extract (1.50 g), which was fractionated with a FCPC-200^®^ instrument (Kromaton Technologies, Angers, France) using a quaternary biphasic solvent system composed of hexane/ethyl acetate/ethanol/water (2:3:2:3, *v*/*v*/*v*/*v*). A pooling fraction (180 mg), mainly containing OLE, was further purified by semi-preparative HPLC using the solvents acetonitrile/acetic acid (99.8:0.2, *v*/*v*, solvent A) and water/acetic acid (99.8:0.2, *v*/*v*, solvent B) under a linear gradient from 20 to 25% for solvent A. As a result, pure OLE (25 mg) was obtained, in which nuclear magnetic resonance (NMR) data (Figures S1 and S2) agreed with those reported in the literature [18].

### 2.4. Synthesis of (−)-Methyl-Oleocanthal (Met-OLE) from (−)-Ligustroside

A wood sample of *Olea europaea* L., obtained from pruning a tree growing in the province of Jaén, Spain, was used to isolate (−)-ligustroside. A sample of wood chips (300 g) was extracted with EtOAc (4 L) for 2 h at reflux. Solvent was evaporated under reduced pressure at 40 °C to give the corresponding dry extract (15 g), which was column chromatographed to give ligustroside (0.34 g), as reported before by the authors [19]. The purity of ligustroside (84%) was determined by an external standard method using the HPLC peak area at 280 nm. Previously, a calibration curve (y = 1734.5 x + 1656; R^2^ = 0.9981) (Graphic S1) was constructed with six standards (0.1–1 mg/mL in MeOH) (Table S1) of pure (−)-ligustroside. For analytical HPLC analyses, separation was carried out by a step gradient with mixtures of MeOH/AcOH (99.8:0.2, *v*/*v*, solvent A) and H_2_O/AcOH (99.8:0.2, *v*/*v*, solvent B). The gradient program consisted of a linear gradient from 20 to 80% A in 55 min, a linear gradient from 80 to 100% A in 5 min and another 10 min to return to initial conditions.

Next, in order to synthetize (−)-methyl-ligustroside, a solution of diazomethane in Et_2_O (35 mL), freshly prepared from *N*-methyl-*N*-nitrosourea (3.5 g) and aq. KOH (50%), was added dropwise to a solution of ligustroside (200 mg) in MeOH (10 mL) and the reaction was left to stir for 60–90 min. The reaction was monitored by TLC and by HPLC using the gradient elution described above. Finally, solvent was removed under reduced pressure to give a brown solid (205 mg). The crude was purified by fast centrifugal partition chromatography (FCPC) using a quaternary biphasic solvent system composed of Hex/EtOAc/EtOH/H_2_O (2:5:2:5, *v*/*v*/*v*/*v*) at a flow of 7 mL/min and a rotation speed of 1200 rpm. As a result, 125 mg (71% yield) of (−)-methyl-ligustroside as a slight yellow–white solid was obtained: ESIMS: *m/z* 561 [M + Na]^+^ (Figure S11); ^1^H NMR (400 MHz, CDCl_3_) (Figure S9): δ (ppm) 7.51 (s, 1H, H-9), 7.15 (m, 2H, H-4′, H-8′), 6.85 (m, 2H, H-5′, 7′), 6.07 (m, 1H, H-5), 5.92 (brs, 1H, H-7), 4.81 (d, 1H, *J*_1″,2″_ = 7.8 Hz, H-1″), 4.25 (dt, 1H, *J*_1′*a*,2′_ = 6.9 Hz, *J*_1′*a*,1′*b*_ = 10.7 Hz, H-1′a), 4.12 (dt, 1H, *J*_1′*b*,2′_ = 6.9 Hz, *J*_1′*b*,1′*a*_ = 10.7 Hz, H-1′b), 3.96 (dd, 1H, *J*_3,2*a*_ = 4.5 Hz, *J*_3,2*b*_ = 9.2 Hz, H-3), 3.89 (dd, 1H, *J*_6″*a*,5″_ = 1.8 Hz, *J*_6″*a*,6″*b*_ = 11.9 Hz, H-6″a), 3.76 (s, 3H, C6′-OCH_3_), 3.71 (s, 3H, C11-OCH_3_), 3.67 (dd, 1H, *J*_6″*b*,5″_ = 5.7 Hz, *J*_6″*b*,6″*a*_ = 11.9 Hz, H-6″b), 3.37 (m, 4H, H-2″, H-3″, H-4″, H-5″), 2.85 (t, 2H, *J*_2′,1′_ = 6.9 Hz, H-2′), 2.70 (dd, 1H, *J*_2*a*,3_ = 4.5 Hz, *J*_2*a*,2*b*_ = 14.2 Hz, H-2a), 2.44 (dd, 1H, *J*_2*b*,3_ = 9.2 Hz, *J*_2*b*,2*a*_ = 14.2 Hz, H-2b), 1.64 (dd, 3H, *J*_6,7_ = 1.5 Hz, *J*_6,5_ = 7.0 Hz, H-6). ^13^C NMR (100 MHz, CDCl_3_) (Figure S10: δ (ppm) 173.1 (C-1), 168.6 (C-11), 159.9 (C-6′), 155.1 (C-9), 131.3 (C-4), 131.0 (C-4′, C-8′), 130.5 (C-3′), 124.8 (C-5), 115.0 (C-5′, C-7′), 109.4 (C-10), 100.8 (C-1″), 95.1 (C-7), 78.5 (C-5″), 77.9 (C-3″), 74.8 (C-2″), 71.5 (C-4″), 66.8 (C-1′a,C-1′b), 62.8 (C-6″a, C-6″b), 55.7 (C6′- OCH_3_), 51.9 (C11-OCH_3_), 41.2 (C-2a, C-2b), 35.1 (C-2′), 31.8 (C-3), 13.5 (C-6). ${\left[\alpha \right]}_{D}^{25}$: −154.0 (*c* 0.7, MeOH). NMR data of this compound agree with those reported in the literature [20]. The purity of (−)-methyl-ligustroside (90%) was determined using the ligustroside’s calibration curve described above.

Finally, to obtain met-OLE, a mixture of methyl-ligustroside (100 mg), water (5 mL) and DMSO (0.4 mL) was subjected to microwave irradiation for 9 min at 180 °C. The reaction was monitored by TLC and HPLC. Then, the crude was extracted with Et_2_O and the solvent was removed under reduced pressure to give a brown oil (58 mg). The purification was achieved by FCPC using Hex:EtOAc:EtOH:H_2_O (1:1:1:1, *v*/*v*/*v*/*v*) as a biphasic solvent system. As a result, 39 mg (64% yield) of met-OLE as a yellow oil was obtained: ESIMS: *m*/*z* 341 [M + Na]^+^ (Figure S7): HRMS (ESI/Q-TOF) *m*/*z* 319.1540 [M + H]^+^ corresponding to C_18_H_22_O_5_ (Figure S8; ^1^H NMR (400 MHz, CDCl_3_) (Figure S3: δ (ppm) 9.63 (brs, 1H, H-8), 9.23 (d, 1H, *J*_9,3_ = 2.0 Hz, H-9), 7.10 (m, 2H, H-4′,H-8′), 6.83 (m, 2H, H-5′,H-7′), 6.62 (q, 1H, *J*_5,6_ = 7.1 Hz, H-5), 4.19 (m, 2H, H-1′), 3.78 (s, 3H, C6′-OCH_3_), 3.61 (m, 1H, H-3), 2.95 (ddd, 1H, *J*_7*b*,8_ = 1.2 Hz, *J*_7*b*,3_ = 8.7 Hz, *J*_7*b*,7*a*_ = 18.3 Hz, H-7b), 2.83 (t, 2H, *J*_2′,1′_ = 7.0 Hz, H-2′), 2.73 (ddd, 1H, *J*_7*a*,8_ = 1.0 Hz, *J*_7*a*,3_ = 5.5 Hz, *J*_7*a*,7*b*_ = 18.3 Hz, H-7a), 2.68 (dd, 1H, *J*_2*b*,3_ = 8.2 Hz, *J*_2*b*,2*a*_ = 15.9 Hz, H-2b), 2.61 (dd, 1H, *J*_2*a*,3_ = 6.7 Hz, *J*_2*a*,2*b*_ = 15.9 Hz, H-2a), 2.07 (d, 3H, *J*_6,5_ = 7.1 Hz, H-6). ^13^C NMR (100 MHz, CDCl_3_): δ (ppm) 200.5 (C-8), 195.2 (C-9), 172.0 (C-1), 158.5 (C-6′), 154.3 (C-5), 143.4 (C-4), 130.0 (C-4′,C-8′), 129.8 (C-3′), 114.1 (C-5′,C-7′), 65.2 (C-1′), 55.4 (C6′-OCH_3_), 46.3 (C-7), 37.0 (C-2), 34.3 (C-2′), 27.4 (C-3), 15.4 (C-6). The full assignment of 1H and 13C NMR resonances was supported by DEPT (Figure S4), HSQC (Figure S5) and HMBC (Figure S6) spectral analyses. ${\left[\alpha \right]}_{D}^{25}$: −1.7 (*c* 1.0, CHCl_3_). The purity of met-OLE (97%) was determined by an external standard method using the HPLC peak area at 280 nm. Previously, a calibration curve (y = 3121.8x − 6706.6; R^2^ = 0.9974) (Graphic S2) was constructed with six standards (0.01–0.5 mg/mL in MeOH) (Table S2) of pure OLE available in authors’ lab.

### 2.5. Animals

Swiss mice were provided by Harlan Interfauna Ibérica^®^ (Barcelona, Spain) and maintained in a temperature-, humidity- and light-controlled room, and were allowed free access to water and food. All animal procedures followed were in accordance with the recommendations of the European Union on animal experimentation (Directive of the European Counsel 2012/707/EU) and were approved by the Animal Ethics Committee of the University of Sevilla (23 July 2018/119).

### 2.6. Murine Macrophages and Spleen Cells Isolation and Culture

Cells were collected 72 h after intraperitoneal sterile thioglycolate injection (3.8% *w*/*v*) as previously described by Montoya et al. (2019) [14]. The collected macrophages were cultured with met-OLE (50, 25, 12.5 μM) pre-treatments for 30 min (min) and then stimulated with LPS from *Escherichia coli* (5 μg/mL) (Sigma-Aldrich^®^, St. Louis, MO, USA) incubating them for 18 h (h) at 5% CO_2_ 37 °C. The supernatants and cells samples were collected and stored at −80 °C until cytokine measurement and Western blotting, respectively.

The mice spleens were harvested and passed through a nylon cell strainer (BD^®^ Biosciences, Franklin Lakes, NJ, USA) with supplemented RPMI1640 medium (10% fetal calf serum, 2 mM glutamine, 1 mM sodium pyruvate, 50 μM 2-mercaptoethanol and 1% penicillin and streptomycin), in order to obtain a cell suspension. Pelleted cells were resuspended in red blood lysis buffer (BD^®^ Biosciences, Franklin Lakes, NJ, USA) and then washed with phosphate buffer solution (PBS). Cells (1 × 10^6^ cells/mL) were left untreated or treated with OLE (50 μM) or, met-OLE (50, 25, 12.5 μM) for 30 min and were LPS-stimulated (5 μg/mL) during an 18 h period at 5% CO_2_, at37 °C. After incubation, cell pellets and supernatants were collected and stored at −80 °C until the quantification of cytokine levels and histone extraction, respectively.

### 2.7. Cell Viability

To evaluate met-OLE cytotoxicity, a sulforhodamine B (SRB) assay was performed [21]. An amount of 1 × 10^5^ cells/mL were cultured in the presence/absence of met-OLE (200–1.6 μM) for 18 h. The absorbance was read at 510 nm with a microplate reader (Biorad^®^, Madrid, Spain). Absorbance is expressed as the percentage of viability when compared to untreated control cells (100% cell survival).

### 2.8. Nitric Oxide Production

Nitrites levels, expressed as the NO generation index, were quantified using Griess reagent in culture supernatants (Sigma-Aldrich^®^, St. Louis, MO, USA), following the protocol reported by Montoya et al. (2018) [22]. A sodium nitrate curve was used as a standard to extrapolate the nitrite amount, and the results were expressed as a percentage compared with DMSO-LPS treated cells (100% nitrites production).

### 2.9. Intracellular ROS Production

The DCDFA assay kit was performed according to the manufacturer’s instructions (Abcam^®^, Cambridge, UK). Cells (2.5 × 10^5^ cells/mL) were seeded on a black plate and then DCDFA (25 μM) was added to each well either previously untreated or treated with met-OLE (50, 25, 12.5 μM) and were LPS-stimulated during 18 h. The results were expressed using H_2_O_2_ (Sigma-Aldrich^®^, Barcelona, Spain) as a positive pro-oxidant control (100% intracellular ROS production), comparing fluorescence intensity [14].

### 2.10. Histone Extraction

Acid extraction was performed as previously reported by Hajji et al. (2010) [5] with brief modifications. The collected spleen cells were washed with PBS, suspended in lysis buffer (10mM Tris pH 6.5, 50 mM sodium bisulfate, 10 nM MgCl_2_, 8.6% sucrose, 1% Triton X-100) and incubated 15 min at 4 °C. After that, the samples were centrifuged at 3500 rpm for 10 min at 4 °C and rewashed. The supernatant was then removed and discarded and Tris–EDTA buffer (10 mM Tris pH 7.4 and 13 mM EDTA) was added to the samples. The precipitated nucleus was resuspended with acid sulfuric 0.2M. After 1 h incubation time, samples were centrifuged at 15,000 rpm for 1 h at 4C, salving supernatants for incubation with acetone overnight at −20 °C. Centrifuged samples were diluted with H_2_O and protein content was measured.

### 2.11. Enzyme-Linked Immunosorbent Assay

IL-6, IFN-γ (Diaclone^®^, Besancon Cedex, France), IL-1β (R&D System^®^, Minneapolis, Cánada, USA), TNF-α and IL-17 (Peprotech^®^, London, UK) concentrations in culture media were measured using specific enzyme-linked immunosorbent assay (ELISA) kits.

### 2.12. Western Blotting

Whole cell lysates, prepared as described by Montoya et al. (2019) [14], and extracted histones were provided into 25 μg protein aliquots. Protein samples were separated by SDS-PAGE (15 or 10%) and electroblotted onto nitrocellulose membranes. Specific primary antibodies were used. The membranes were then incubated with the corresponding secondary antibody for 2 h. The results were obtained from at least six independent experiments. A chemiluminescence light detection kit (Pierce^®^, Rockford, IL, USA) and Amersham Imager 600 equipment (GE Healthcare^®^, Chicago, IL, USA) were used for the detection of immunosignals. Data were normalized with a housekeeping control and quantified by Image Processing and Analysis in Java (Image J^®^, Bethesda, MD, USA).

### 2.13. Statistical Analysis

Data in figures and text are reported as arithmetic means ± standard error (SEM) from at least six independent experiments carried out in triplicate. Results were evaluated using Graph Pad Prism version 5.01 software (San Diego, CA, USA), analyzing the statistical significance by a one-way analysis of variance (ANOVA), followed by the Tukey’s multiple comparisons test. The *p*-values < 0.05 were considered statistically significant. Figures from densitometry experiments are representatives of different experiments performed on a different day.

## 3. Results

### 3.1. Chemistry

**Figure 1:**
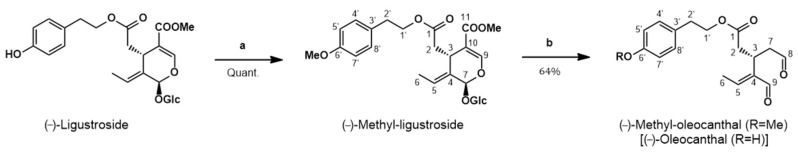
Synthesis of (−)-methyl-oleocanthal from (−)-ligustroside. Reagents and conditions: (**a**) CH_2_N_2_/Et_2_O; (**b**) H_2_O-DMSO, MW, 180 °C, 9 min.

The synthesis of (−)-met-OLE was carried out herein from (−)-ligustroside (Figure 1), inspired by the reaction reported by Skaltsounis’ group for the conversion of oleuropein (same structure as ligustroside but with an additional OH at C-5′) into oleacein (same structure as OLE but with an additional OH at C-5′) [23]. These authors achieved the semi-synthesis of oleacein from oleuropein under Krapcho decarbomethoxylation conditions in one single step. They refluxed oleuropein, dissolved in wet DMSO, with two equivalents of an inorganic salt (NaCl) for 10 h to obtain oleacein with a 21% yield, after purification by silica gel column chromatography. Procopio’s group later improved this attractive semi-synthesis through changing DMSO by water and heating in a microwave reactor to get oleacein in just 20 min with a 48% yield [24]. Our group was also working to improve the semi-synthesis of oleacein and OLE from oleuropein and ligustroside, respectively, following the recommendations of a previous work aimed at adapting the classical Krapcho decarboxylation experimental conditions to an aqueous microwave scenario [25], when Procopio’s work came to light (unpublished results).

Thus, our synthesis of met-OLE (Figure 1) started with the isolation of ligustroside from olive wood following a procedure previously reported by us [19]. Then, ligustroside was methylated using diazomethane, prepared in situ from *N*-methyl-*N*-nitrosourea. The conversion of ligustroside into methyl-ligustroside was quantitatively performed at room temperature for around 1 h. If the starting material (ligustroside) had been pure enough, methyl-ligustroside would have been sufficiently pure to be used directly in the next step. However, ligustroside was isolated with a purity of 84% from the olive wood extract and it was convenient to submit crude methyl-ligustroside to purification, in order to eliminate the minor components that accompanied the starting material (ligustroside). On this occasion, we used fast centrifugal partition chromatography (FCPC) to get adequately pure methyl-ligustroside in one single run. Then, pure methyl-ligustroside was submitted to the Krapcho reaction under microwave irradiation. Several attempts were carried out, with varying temperatures, reactions time and solvent ratios, until the best conditions were found. Thus, the reaction of methyl-ligustroside in water, with the minimum amount of DMSO to dissolve the substrate, was performed in a microwave reactor for 9 min at 180 °C, reaching the complete conversion of methyl-ligustroside into met-OLE. It is worth noting that this Krapcho reaction took place in the absence of inorganic salt, as Murphree’s group [25] came to observe in some cases and as Fernández-Bolaños’ group has recently claimed [26].

In order to purify the final compound and taking into account the known sensitivity of related OLE and oleacein to decomposition when in contact with solid stationary phases, such as silica gel [27], and upon exposure to oxygen and light [12], we used the FCPC technique for the purification of met-OLE. This allowed us to obtain pure met-OLE with the same success as when we previously purified OLE from an olive oil phenolic extract [18]. This silica-free chromatographic technique has shown to be a good option to isolate these compounds [28] and consequently improve the yield of the reaction step. Thus, we have performed the conversion of methyl-ligustroside into met-OLE in a 64% yield, which is the best yield reported to date for the Krapcho conversion of ligustroside/oleuropein or their derivatives into the corresponding dialdehydes. Met-OLE has been synthesized in this work for the first time.

### 3.2. Effects of Met-OLE on Cell Viability

**Figure 2:**
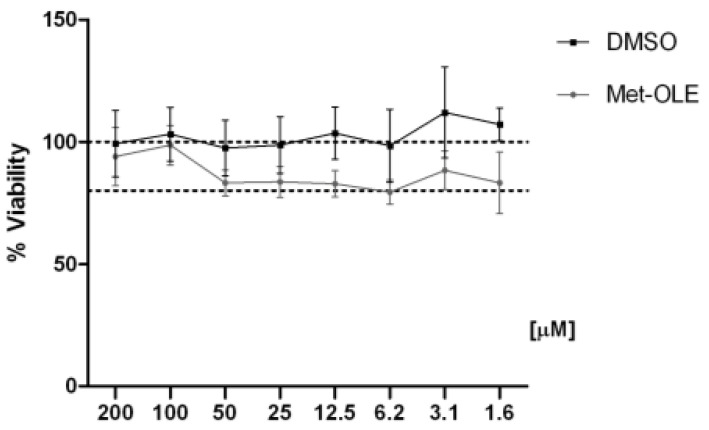
Effect of met-OLE on cell viability. Macrophages were pretreated with met-OLE (200–1.6 μM) for 18 h. Cell survivals were expressed as the percentage of viability with respect to 100% from control, untreated cells. Results are presented as mean ± SEM of at least six independent experiment.

First, we evaluated the cell viability after met-OLE treatments using a SRB assay. Data show that met-OLE was not cytotoxic after 18 h of treatment with concentrations of 1.6 up to 200 μM and did not compromise the cell viability significantly (≥80%) (Figure 2). Therefore, based on our previous work using OLE [14], we selected 12.5, 25 and 50 μM concentrations of met-OLE to be studied in the following assays.

### 3.3. Effects of Met-OLE on IL-1β, IL-6, IL-17, IFN-γ and TNF-α Production

**Figure 3:**
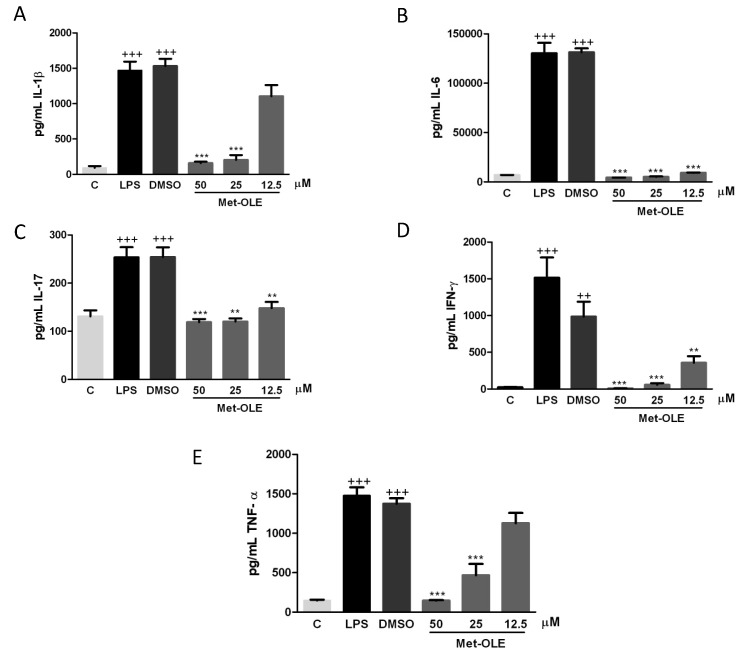
Pro-inflammatory cytokine levels were down-regulated in met-OLE treated cells. Macrophages were pretreated with met-OLE (50, 25 or 12.5 μM) for 30 min and then, LPS-stimulated during 18 h. (**A**) IL-1β, (**B**) IL-6, (**C**) IL-17, (**D**) IFN-γ and (**E**) TNF-α levels were measured by ELISA on cell supernatants. Results are presented as the mean ± SEM of at least six independent experiments. ++ *p* < 0.01; +++ *p* < 0.001 vs. unstimulated control cells; ** *p* < 0.01; *** *p* < 0.001 vs. LPS-DMSO stimulated cells.

To explore the effects of met-OLE on pro-inflammatory cytokine production, we evaluated IL-1β, IL-6, IL-17, IFN-γ and TNF-α levels. As shown in Figure 3, after 18 h of exposure to LPS, murine cells exhibited higher levels of pro-inflammatory cytokines than unstimulated control cells (++ *p* < 0.01; +++ *p* < 0.001 vs. unstimulated cells). On the contrary, when cells were treated with met-OLE, we observed a significantly down-regulation of IL-1β, IL-6, IL-17, IFN-γ and TNF-α secretions when compared to the LPS-DMSO group (** *p* < 0.01; *** *p* < 0.001 vs. LPS-DMSO stimulated cells).

### 3.4. Effects of Met-OLE on Intracellular ROS and NO Productions

In terms of elucidating the role of met-OLE in the oxidative and inflammatory response mediated by LPS, we measured intracellular ROS and NO levels using DCFDA and Griess assays, respectively, in LPS-induced murine peritoneal macrophages.

**Figure 4:**
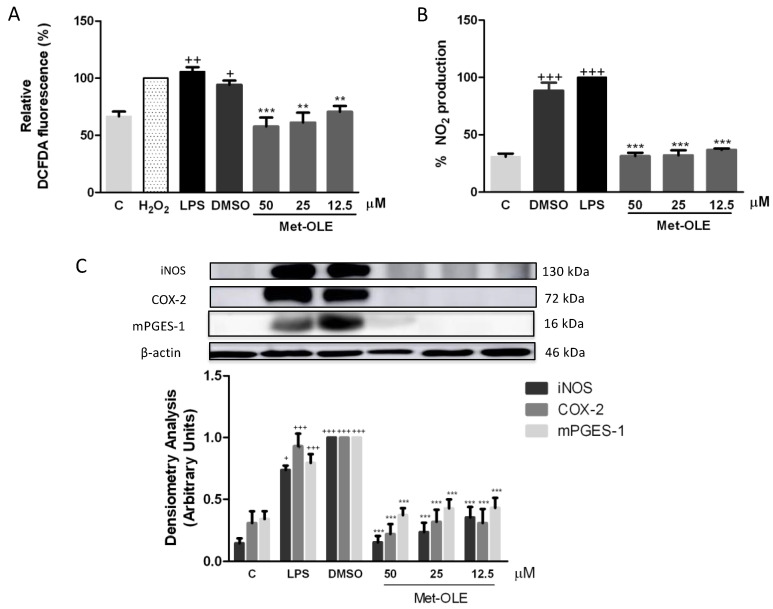
Effects of met-OLE on (**A**) intracellular ROS and (**B**) NO production and (**C**) overexpression of the iNOS, COX-2 and mPGES-1 proteins. Macrophages were pretreated with met-OLE (50, 25 or 12.5 μM) for 30 min and then, LPS-stimulated during 18 h. Subsequently, ROS and NO levels were measured using DCFDA and Griess assays in supernatants, respectively. The expression of iNOS, COX-2 and mPGES-1 proteins was then measured in whole cell lysates and analyzed by Western blotting. Results are presented as mean ± SEM of at least six independent experiments. + *p* < 0.05; ++ *p* < 0.01; +++ *p* < 0.001 vs. unstimulated control cells; ** *p* < 0.01; *** *p* < 0.001 vs. LPS-DMSO stimulated cells.

Data revealed remarkable overproductions of ROS and NO induced by LPS in murine macrophages when compared to unstimulated cells (+ *p* < 0.05; ++ *p* <0.01; +++ *p* < 0.001 vs. unstimulated control cells) (Figure 4A,B). Meanwhile, levels of both mediators were significantly reduced after met-OLE treatments (** *p* < 0.01; *** *p* < 0.001 vs. cells stimulated cells).

### 3.5. Met-OLE Down-Regulated iNOS, COX-2 and mPGES-1 Protein Overexpression Induced by LPS in Murine Macrophages

To investigate whether the effects of met-OLE on NO accumulation were associated with the expression of the iNOS protein, we performed an immunoblotting assay with cell lysates. Consistently, cells from secoiridoid-related phenolic groups showed decreased iNOS protein overexpression when compared to those of the LPS-DMSO exposed group (*** *p* < 0.001 vs. LPS-DMSO stimulated cells) (Figure 4C).

In addition, to gain further insight into the anti-inflammatory potential of met-OLE, we studied its effects on COX-2 and PGE_2_ related biomarkers. As expected, after LPS exposure, COX-2 and mPGES-1 expressions increased significantly (+++ *p* < 0.001 vs. unstimulated control cells). On the contrary, met-OLE pretreatments effectively counteracted induction of the expressions of these proinflammatory enzymes (*** *p* < 0.001 vs. LPS-DMSO stimulated cells) (Figure 4C).

### 3.6. Effects of Met-OLE on LPS-Induced MAPKs Activation in Murine Peritoneal Macrophages

**Figure 5:**
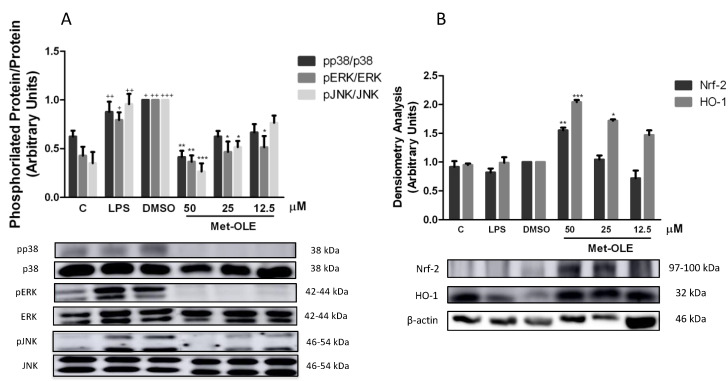
Met-OLE inhibited (**A**) MAPKs phosphorylation and (**B**) upregulated the Nrf-2/HO-1 antioxidation axis in LPS-induced cells. Macrophages were pretreated and stimulated with LPS during 18 h with met-OLE (50, 25 or 12.5 μM). Subsequently, MAPKs, Nrf-2, and HO-1 protein expressions were measured in whole cell lysates by Western blot. Results are presented as the mean ± SEM of at least six independent experiments. + *p* < 0.05; ++ *p* < 0.01; +++ *p* < 0.001 vs. unstimulated control cells; * *p* < 0.05; ** *p* < 0.01; *** *p* < 0.001 vs. LPS-DMSO stimulated cells.

Murine macrophages were previously pretreated with met-OLE and were LPS-induced. After 18 h, cells from the LPS-DMSO control group showed a marked phosphorylation of MAPK members: p38, ERK and JNK (+ *p* < 0.05; ++ *p* < 0.01; +++ *p* < 0.001 vs. unstimulated cells). Nevertheless, met-OLE counteracted the activation of these proteins and exhibited a lower phosphorylation for p38, ERK and JNK MAPK (* *p* < 0.05; ** *p* < 0.01; *** *p* < 0.001 vs. LPS-DMSO stimulated cells (Figure 5A).

### 3.7. Effects of Met-OLE on Nrf2-Mediated Transcriptional Activation and HO-1 Induction in LPS Murine Peritoneal Macrophages

To identify whether the OLE derivative evoked an antioxidant effect on cells, we studied the Nrf-2/HO-1 axis, measuring both protein expressions by immunoblotting. As shown in Figure 5B, Nrf-2 and HO-1 protein expression was significantly up-regulated in met-OLE-exposed cells when compared to LPS-DMSO controls (* *p* < 0.05; ** *p* < 0.01; *** *p* < 0.001 vs. LPS-DMSO stimulated cells). The expression of the antioxidant Nrf-2/HO-1 protein that was induced was according to the ROS reduction observed in Figure 4A. Altogether our data highlighted the high antioxidant potential of met-OLE.

### 3.8. Effects of Met-OLE on Canonical and Noncanonical Inflammasome Signaling Pathways

**Figure 6:**
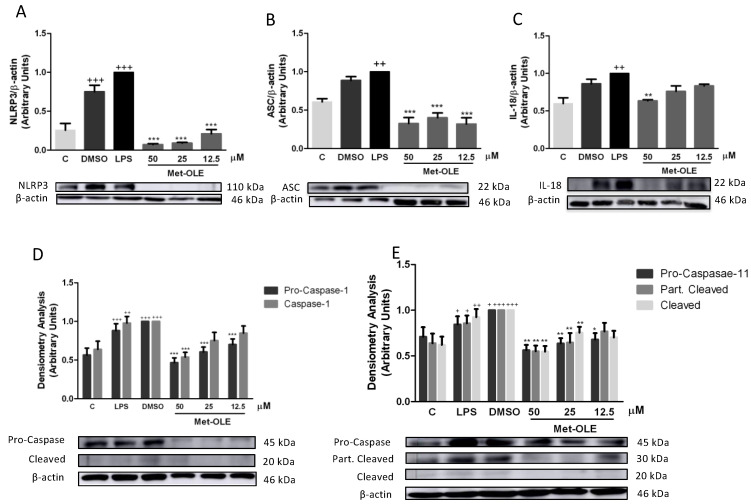
Met-OLE modulated the canonical and noncanonical inflammasome signaling pathways. Cells were pretreated with met-OLE (50, 25 or 12.5 μM) for 30 min and then, LPS-stimulated during 18 h. (**A**) NLRP3, (**B**) ASC, (**C**) IL-18, (**D**) Pro- and caspase-1 and (**E**) Pro-, partially cleaved and cleaved caspase-11 protein expressions were evaluated by Western blot. Results are presented as the mean ± SEM of at least six independent experiments. + *p* < 0.05; ++ *p* < 0.01; +++ *p* < 0.001 vs. unstimulated cells; * *p* < 0.05; ** *p* < 0.01; *** *p* < 0.001 vs. LPS-DMSO stimulated cells.

In the presence of LPS, the inflammasome induces the inflammatory response in murine peritoneal macrophages [14,21]. Unsurprisingly, after 18 h of LPS exposition, cells showed a marked induction of canonical and noncanonical inflammasome implicates (+ *p* < 0.05; ++ *p* < 0.01; +++ *p* < 0.001 vs. unstimulated cells). According to the canonical pathway, we observed that met-OLE modulated the protein levels of nucleotide-binding oligomerization domain-like receptor protein 3 (NLRP3) and apoptosis-associated speck-like protein containing a caspase recruitment domain (ASC), which activated pro-caspase-1 and caspase-1. All of these inflammasome canonical pathway proteins were effectively negatively regulated in pretreated macrophages (Figure 6).

The protein expressions of the non-canonical pathway were then analyzed. As shown in Figure 5E, the expressions of pro-caspase-11, partially cleaved and cleaved caspase-11 were significantly reduced in cells incubated with met-OLE incubated cells (* *p* < 0.05; ** *p* < 0.01; *** *p* < 0.001 vs. LPS-DMSO stimulated cells). Lastly, in accordance with previous results, the expression levels of the mature form of IL-18 were measured, showing a significant reduction after met-OLE 50 μM treatment (** *p* < 0.01 vs. LPS-DMSO stimulated cells) (Figure 6C).

Collectively, met-OLE exhibited significant regulatory effects in LPS-induced canonical and non-canonical inflammasome signaling pathways in murine peritoneal macrophages.

### 3.9. OLE and Met-OLE Induced Epigenetic Histone Modifications

**Figure 7:**
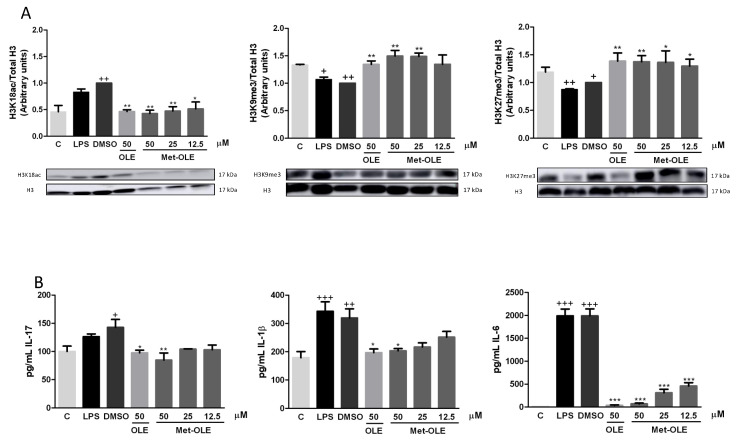
(**A**) Epigenetic modifications of histones by OLE and met-OLE and (**B**) the cytokine-related response. Spleen cells were pre-treated with OLE (50 μM) or met-OLE (50, 25 or 12.5 μM) for 30 min and then, LPS-stimulated during an 18 h period. The histones were isolated from cells and the expression of H3 modifications was evaluated using antibodies for H3K18ac, H3K9me3 and H3K27me3 by Western blot. Related cytokine levels were measured in cell supernatants using specific ELISA assays. Results are presented as the mean ± SEM of at least six independent experiments. + *p* < 0.05; ++ *p* < 0.01; +++ *p* < 0.001 vs. unstimulated cells; * *p* < 0.05; ** *p* < 0.01; *** *p* < 0.001 vs. LPS-DMSO stimulated cells.

In an attempt to elucidate the role of epigenetic histone mechanisms underlying met-OLE’s anti-inflammatory effects, we explored met-OLE-induced epigenetic changes in histone markers and cytokine-correlated production, compared to OLE. We isolated histones from mice spleen cells and measured their expression by Western blot. A total of three H3 histone modifications were evaluated on several lysine residues: H3K18ac, H3K9me3 and H3K27me3. Acetylation of H3 is associated with the induction of expression, while methylation is a repressive marker. Consistent with that, basal levels of H3K18ac were induced, as well as H3K9me3 and H3K27me3 being significantly decreased after exposure to LPS exposition (+ *p* < 0.05; ++ *p* < 0.01 vs. unstimulated cells). Surprisingly, as shown in Figure 7A, pretreated cells with OLE and met-OLE counteracted LPS induction, preventing H3K18 acetylation or H3K9 and H3K27 demethylation (* *p* < 0.05; ** *p* < 0.01 vs. LPS-DMSO stimulated cells). Subsequently, we next evaluated the levels of several modulated cytokines that have been associated with the markers of these histones in their promoter regions. Particularly, levels of IL-17, IL-1β and IL-6 were up-regulated after LPS stimulation (+ *p* < 0.05; ++ *p* < 0.01; +++ *p* < 0.001 vs. unstimulated cells), while the treatments with OLE and met-OLE were able to considerably reduce them. These results with previous results obtained for the histones’ modifications (* *p* < 0.05; ** *p* < 0.01; *** *p* < 0.001 vs. LPS-DMSO stimulated cells) (Figure 7B).

Interestingly, all together the results suggest that OLE and met-OLE exert effects on epigenetic regulation in acute inflammatory responses and innate immunity, reducing H3K18 acetylation and inducing H3K9 and H3K27 methylation on *il17, il1β* and *il6* promoters, and probably suppressing their transcription.

## 4. Discussion

In the search for new strategies to improve the chemistry and metabolic stability of some compounds, methylation has been postulated as a novel approach. This fact is supported by several studies that reported how phenolic hydroxyl group methylation (orthomethylation) generated more lipophilic molecules, promoting the induction of beneficial effects when compared to its parent non-methylated analogs [29,30]. During et al. (2013) compared the effects of Chrysin, a natural flavone, and its methyl derivative in a Caco-2 cell model. They observed that methylation improved the inhibition of IL-18, IL-6, COX-2 and NF-κB expression in a better way than Chrysin [29]. In addition, another in vitro study on RAW264.7 LPS-stimulated cells showed the inhibitory potential of two resveratrol methyl-derivates that blocked the MAPKs and NF-κB signaling pathways [2]. Altogether, these works suggest that the orthomethylated form of polyphenols has a better metabolic and biological profile, as well as bioactivity, than its nonmethylated patterns, so it could confer a viable solution to the bioavailability and biotransformation deficiencies of secoiridoids and, even better, improve its pharmacological effects. Our data provided evidence that methylation of the OLE hydroxyl group appears to enhance its pharmacodynamic and pharmacological profile.

Excess ROS production is associated with the production and release of inflammatory mediators. Similarly, numerous studies have reported an increase in COX-2, iNOS, and NO-related secretion in the LPS-induced inflammatory response [14,22,31]. In fact, iNOS is predominantly overexpressed in LPS-induced macrophages and high levels of NO may also contribute to the inflammatory process. In this line, inhibition of NO synthesis has been proposed to be a possible mechanism of action of drugs used to treat inflammatory diseases. In addition, COX-2 is one of the most important mediators of the inflammatory response, since it is responsible for the synthetization of prostaglandins. First, COX-2 catalyzes arachidonic acid PGH2 production and then, mPGES-1 converts it to PGE2. Our findings have shown for the first time that the OLE metabolite, met-OLE, reduced intracellular ROS production, as seen in the nitrates/nitrites levels, which are indicators of NO production, as well as the expression of iNOS, COX-2 and mPGES-1 proteins. Interestingly, met-OLE-treated cells exerted a more significant down-regulation when compared to the parent compound in LPS-induced murine peritoneal macrophages [14].

An imbalance of pro-inflammatory Th1 and Th17 cytokines is associated with macrophage LPS exposure. This fact is reflected in several reports, where LPS-activated cells showed higher levels of certain cytokines than non-stimulated cells [32,33]. In agreement with these observations, the present results revealed that IL-1β, IL-6, IL-17, TNF-α and IFN-γ levels were increased after 18 h of LPS activation, while met-OLE treatment significantly ameliorated all these cytokines’ levels, reducing, even more efficiently, TNF-α and IL-17 secretion levels when compared to OLE [14]. Therefore, regulation of these pro-inflammatory biomarkers could represent a potential molecular target that is susceptible to met-OLE modulation, which has not been demonstrated previously.

LPS-induced signaling pathways include MAPKs, a subfamily of serine/threonine-specific protein kinases family whose phosphorylation is significantly induced by LPS in macrophages, initiating inflammatory gene expression, including, in particular, iNOS and COX-2. MAPKs include three main members: p38, JNK and ERK. In this sense, inhibition of p38 MAPK has been shown to effectively alleviate inflammatory diseases, including rheumatoid arthritis, asthma, cardiovascular disease, Alzheimer’s disease, osteoarthritis and inflammatory pain [34]. Thus, MAPKs inhibitors are considered novel potential drug candidates for inflammation-related diseases. In the present study, we reported that met-OLE suppressed MAPKs’ phosphorylation in a concentration-dependent manner. These results are in line with those from our previous studies, which reported that MAPKs’ activation was drastically inhibited by OLE in LPS -induced murine macrophages [14], as well as in the paws of arthritic animals fed with this EVOO phenol [15].

In contrast, activation of the antioxidant Nrf-2/HO-1 pathway exerts protective actions in LPS-induced peritoneal macrophages or RAW264.7 murine macrophages through down-regulation of pro-inflammatory mediators, such as iNOS, COX-2, NO, PGE2 or multiple cytokines [14,22,35]. The Nrf-2/HO-1 pathway in activated macrophages is an attractive target, due to its protective effect against ROS-induced cell damage. Moreover, HO-1 expression can inhibit the production of TNF-α, IL-1β and IL-6 cytokines in RAW264.7 macrophages [35]. Here, we describe that met-OLE -treated cells restored Nrf-2 and HO-1 LPS-induced protein overexpression, conferring a notable role of the Nrf-2/HO-1 signaling pathway in the beneficial effects of met-OLE in acute inflammatory response. Furthermore, these results were consistent with the decrease in the production of inflammatory markers described above.

Inflammasomes are multimolecular complexes that can sense the presence of a pathogen and trigger a robust pro-inflammatory response. In particular, it is involved in the innate immune response, where it participates in host defense, cancer and autoinflammation, among others [36]. The inflammasome complex consists of a sensor (NLRP3), an adaptor (ASC) and a zymogen (pro-caspase-1). The outcome of its induction is maturation of IL-1β and IL-18 cytokines and consequently pyroptosis, an inflammatory cell death. It is well documented that in the presence of LPS, oxidative stress, certain cytokines or NF-kB and MAPKs signaling activation, NLRP3 may be induced. Then, assembly in the canonical way is performed up to cytokine secretion and pyroptosis [37]. Furthermore, a non-canonical inflammasome pathway has been described as an alternative mechanism to induce pyroptosis without intermediates and caspase-1-dependent maturation of IL-1β and IL-18 cytokines [37]. In our study, LPS-induced inflammation led to activation of the canonical cascade with caspase-11 cleaved, which was accompanied by increased pro-inflammatory cytokine production. In contrast, met-OLE treatment was able to significantly return these effects to their basal levels. These findings are consistent with our previous study, where OLE inhibited the canonical and non-canonical inflammasome signaling pathways [14]. However, it is important to note that our finding reveals, for the first time, a remarkable role for met-OLE, in inflammasome blockade, showing even a more significant down-regulation of inflammasome-activated protein expression in comparison with its parent compound, OLE.

Identifying factors involved in the onset and progression of inflammation and the discovery of epigenetic modifiers are essential to better understand the pathogenesis of inflammatory diseases and the search for therapeutic targets. Epigenetic alterations are suggested to be responsible for disease-related phenotypic differences in unaltered DNA sequences [38]. Certain dietary habits appear to influence histone modifications, including methylation, acetylation, and phosphorylation, among others [38,39]. Particularly, the epigenetic and epigenomic effects described by food components of the Mediterranean diet that display positive health effects against several diseases include the modulation of miRNA expression and histone modifications [40].

According to EVOO, one of the primary fat sources in the Mediterranean diet, only one study investigated EVOO-induced histone modifications in association with anti-inflammatory activity. EVOO was able to restore histone deacetylase (HDAC) 1 and HDAC3 overexpression levels induced by low-level inflammation in the THP-1 cell line [9]. Additionally, various studies showed the ability of olive oil polyphenols, such as oleuropein and oleacein, to decrease HDACs, but these results have been described in terms of their anticancer effects.

In this line, we evaluated, for the first time, the effects of met-OLE in comparison with OLE on LPS-induced histone modifications in H3K9, H3K27 and H3K18. Our results suggested that LPS induced H3 acetylation or methylation and resulted in sustained IL-1β, IL-6 and IL-17 expression in immune spleen cells. Accordingly, Imuta et al. (2020) observed H3K27 demethylation on the *il1b* promoter, which was associated with an increase of IL-1β levels on LPS- and IFN-stimulated RAW264.7 cells [7]. Other in vitro assays showed a marked reduction in H3K9me3 levels using LPS-exposed murine macrophages that was related to NF-κB recruitment, activating IL-6 transcription [6,8]. Although histone H3K9 and H3K27 methylation could promote different roles based on immune cell type, metabolic status, target gene, stimulus and time of exposition [41], H3K9me3 and H3K27me3 are associated with chromatin condensation and, therefore, gene repression [42]. On the contrary, H3K18 acetylation assists in the accessibility of DNA machinery transcription, inducing pro-inflammatory cytokine gene transcription [43]. Consistent with all of these authors, our data suggested that H3K27me3, H3K9me3 and H3K18ac were involved in the expression of pro-inflammatory cytokines induced by LPS. However, in the presence of OLE and met-OLE, these histone markers were properly modulated to control IL-1β, IL-6 and IL-17-related production in immune spleen cells.

In fact, inappropriate histone methylation can cause inflammation and damage. Recently, emerging studies have demonstrated the regulatory roles of histone methyltransferases in inflammasome activation, suggesting that they also cooperate with inflammasomes and play regulatory roles in the inflammasome-induced inflammatory response [44]. Furthermore, increasing evidence has evaluated the association between histone H3 acetylation and the NLRP3 inflammasome. The direct relationship between the high level of H3 acetylation in the promoter region of NLRP3 has been widely established in different in vitro and in vivo studies related to several pathologies [45,46,47,48]. Sun et al. (2017) also demonstrated that by using curcumin, an inhibitor of histone acetylation, NLRP3 activation was prevented [46]. Taking this into account, the results obtained in the evaluation of NLRP3 and, subsequently, caspase-1 activation suggest a potential downstream consequence of histone acetylation. Therefore, the present study revealed, for the first time, that OLE and met-OLE may exert a critical role in inflammasome blockade through modifications of histone epigenetics, regulating the expression of immune and inflammatory-related genes.

## 5. Conclusions

Collectively, this study reports, for the first time, the semi-synthesis of a new methylated OLE metabolite and its antioxidant and anti-inflammatory effects in the response of murine macrophages to LPS exposure. Generally, met-OLE inhibited the expression of pro-inflammatory enzymes, (iNOS, COX-2 and mPGES-1), cytokines (IL-6, IL-17, IL-1β, TNF-α, IFN-γ and IL-18) and regulated intracellular ROS and products that are related to oxidative damage, such as NO levels, via MAPKs, Nrf-2/HO-1 and both canonical and non-canonical inflammasome signal pathway modulation. Furthermore, both OLE and met-OLE were able to regulate epigenetic mechanisms by modulating histone methylation (H3K9me3 and adnH3K27me) and acetylation (H3K18ac) and by down-regulating cytokine-related production in LPS-exposed spleen immune cells.

This revealing evidence suggests that the methylated metabolite of OLE may contribute significantly to the beneficial effects that are associated with the secoiridoid-related compound and the usual consumption of EVOO. Thus, met-OLE could be a promising therapeutic agent used in various immune—inflammatory pathologies, and the elucidation of their structure—activity relationship might be a relevant goal for future directions.

## Data Availability

Data is contained within the article.

## Supplementary Materials

Supplementary materials are available online https://www.mdpi.com/article/10.3390/antiox11010056/s1, synthesis of diazomethane’s precursor; Western Blotting; Calibration curves: Graphics S1 and S2, Tables S1 and S2; Spectroscopy data of OLE (Figures S1 and S2), met-OLE (Figures S3–S8) and methyl-ligustroside (Figures S9–S11).
