# Supplementary material for: (−)-Methyl-Oleocanthal, a New Oleocanthal Metabolite Reduces LPS-Induced Inflammatory and Oxidative Response: Molecular Signaling Pathways and Histones Epigenetic Modulation
Source: Antioxidants (Basel). 2021 Dec 27;11(1):56. doi: 10.3390/antiox11010056 (PMC8772879; doi:10.3390/antiox11010056)
Supplement: Supplementary file 1 [file antioxidants-11-00056-s001.zip › antioxidants-1516591-supplementary.pdf]

Supplementary materials

Table of contents

1. Synthesis of diazomethane's precursor
2. Western Blotting
3. Calibration curves
4. Spectroscopy data of OLE, met-OLE and methyl-ligustroside

1. Synthesis of diazomethane's precursor

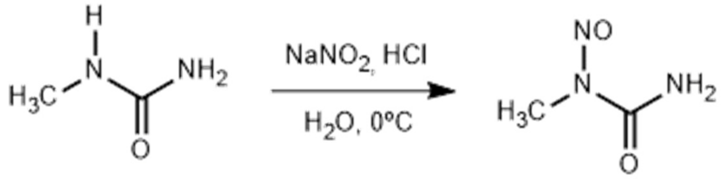

The synthesis of diazomethane's precursor was achieved following a reported procedure (Huang *et al.*, 2016). A mixture of *N*-methylurea (14.8 g), $NaNO_2$ (15.2 g) and water (120 mL) was stirred until dissolution of products. Next, the mixture was cooled to 0 ºC and concentrated HCl (26 mL) was added dropwise for 1 h. The mixture was left to stir for 30 minutes to 0 ºC. Finally, the resulting precipitate was filtered, washed with cooled water (20 mL), and dried under vacuum. As a result, a beige solid was obtained (14.4 g, 70% yield).

Huang, Z.; Huang, X.; Li, B.; Mou, C.; Yang, S.; Song, B.A.; Chi, Y.R. Access to P-stereogenic phosphinates via *N*-heterocyclic carbene-catalyzed desymmetrization of bisphenols. *J. Am. Chem. Soc.* **2016**, *138*, 7524–7527, doi: 10.1021/jacs.6b04624.

2. Western Blotting

Antibody	Dilution	Source
**iNOS**	1:1000	Cell Signaling Technology® (Danvers, MA, USA)
**COX-2**	1:1000	Cell Signaling Technology® (Danvers, MA, USA)
**mPGES-1**	1:1000	Abcam® (Cambridge, UK)
**p-P38**	1:1000	Cell Signaling Technology® (Danvers, MA, USA)
**P38**	1:1000	Cell Signaling Technology® (Danvers, MA, USA)
**p-ERK**	1:1000	Cell Signaling Technology® (Danvers, MA, USA)
**ERK**	1:1000	Cell Signaling Technology® (Danvers, MA, USA)
**p-JNK**	1:1000	Cell Signaling Technology® (Danvers, MA, USA)
**JNK**	1:1000	Cell Signaling Technology® (Danvers, MA, USA)
**Nrf-2**	1:1000	Cell Signaling Technology® (Danvers, MA, USA)
**HO-1**	1:1000	Enzo Life Sciences® (Inc. Farmingdale, NY, USA)
**NLRP3**	1:1000	Cell Signaling Technology® (Danvers, MA, USA)
**ASC**	1:1000	Cell Signaling Technology® (Danvers, MA, USA)
**IL-18**	1:1000	Abcam® (Cambridge, UK)
**Caspase-1**	1:1000	Novus Biologicals®, Littleton, CO, USA
**Caspase-11**	1:1000	Novus Biologicals®, Littleton, CO, USA
**H3**	1:1000	Cell Signaling Technology® (Danvers, MA, USA)
**H3K18ac**	1:1000	Cell Signaling Technology® (Danvers, MA, USA)
**H3K27me3**	1:1000	Cell Signaling Technology® (Danvers, MA, USA)
**H3K9me3**	1:1000	Cell Signaling Technology® (Danvers, MA, USA)
**β-Actin**	1:2500	Abcam® (Cambridge, UK)

3. Calibration curves

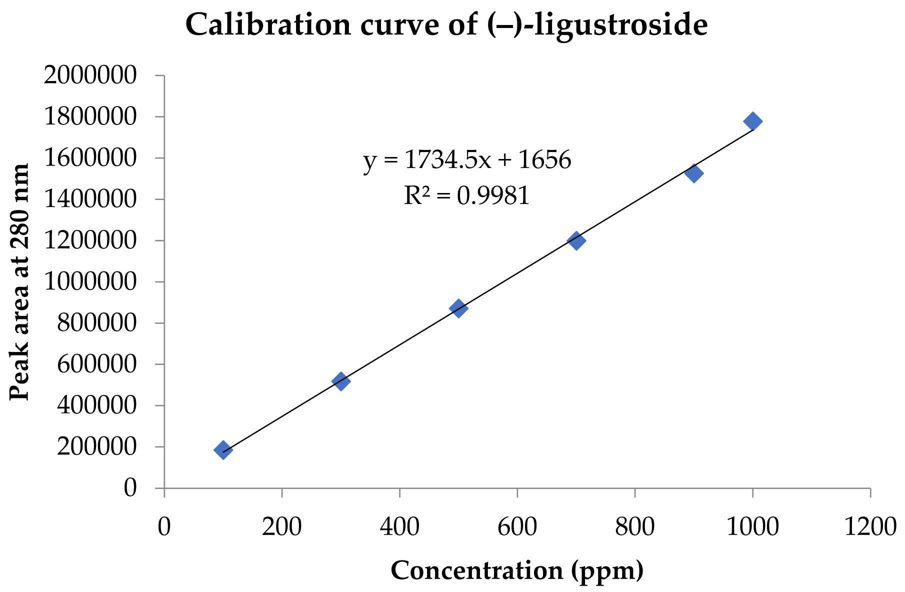

Graphic S1. Peak area at 280 nm vs (–)-ligustroside concentration (ppm).

Table S1. Concentration of prepared solutions of (–)-ligustroside and their peak area values at 280 nm.

(–)-Ligustroside concentration (ppm)	Peak area at 280 nm
100	185383
300	518516
500	871968
700	1200114
900	1526396
1000	1778304

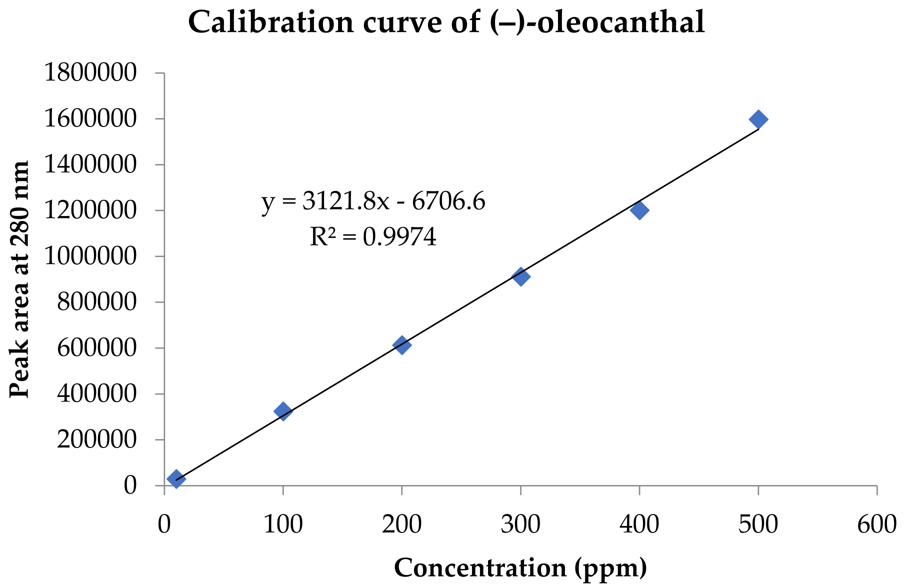

Graphic S2. Peak area at 280 nm vs (–)-oleocanthal concentration (ppm).

Table S2. Concentration of prepared solutions of (–)-oleocanthal and their peak area values at 280 nm.

(–)-Oleocanthal concentration (ppm)	Peak area at 280 nm
10	28987
100	323681
200	612313
300	911136
400	1200596
500	1596912

4. Spectroscopy data of OLE, met-OLE and methyl-ligustroside

(–)-Oleocanthal (**OLE**)

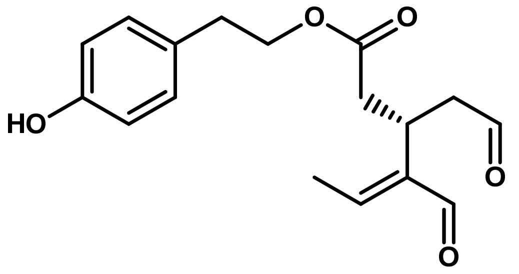

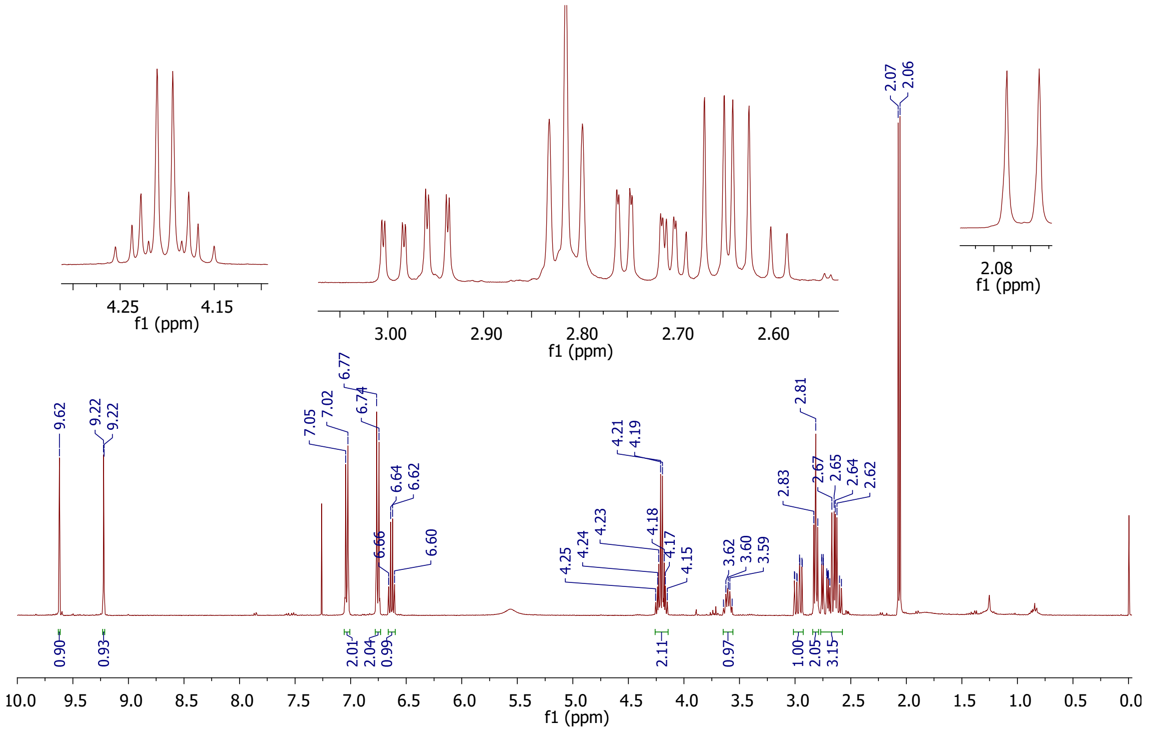

Figure S1. ^{1}H NMR of (–)-oleocanthal in deuterated chloroform.

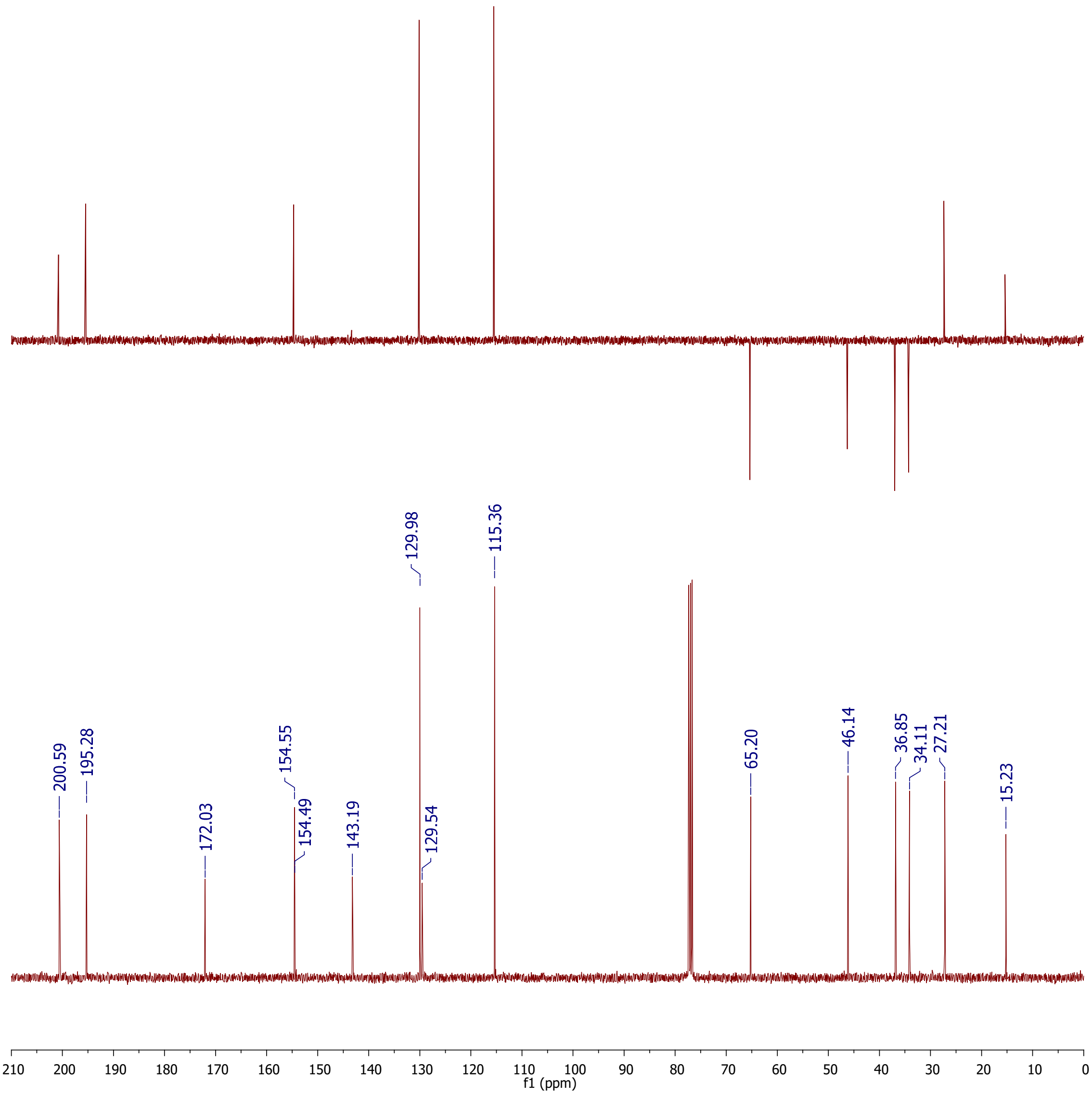

Figure S2. ^{13}C NMR (bottom) and Dept-135 (top) of (–)-oleocanthal in deuterated chloroform.

(–)-Methyl-oleocanthal (**Met-OLE**)

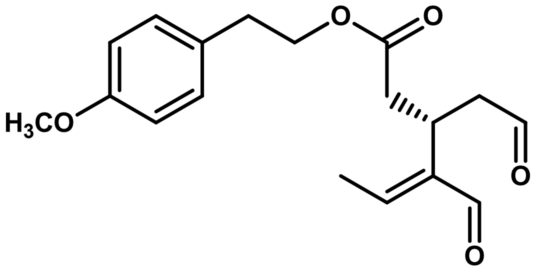

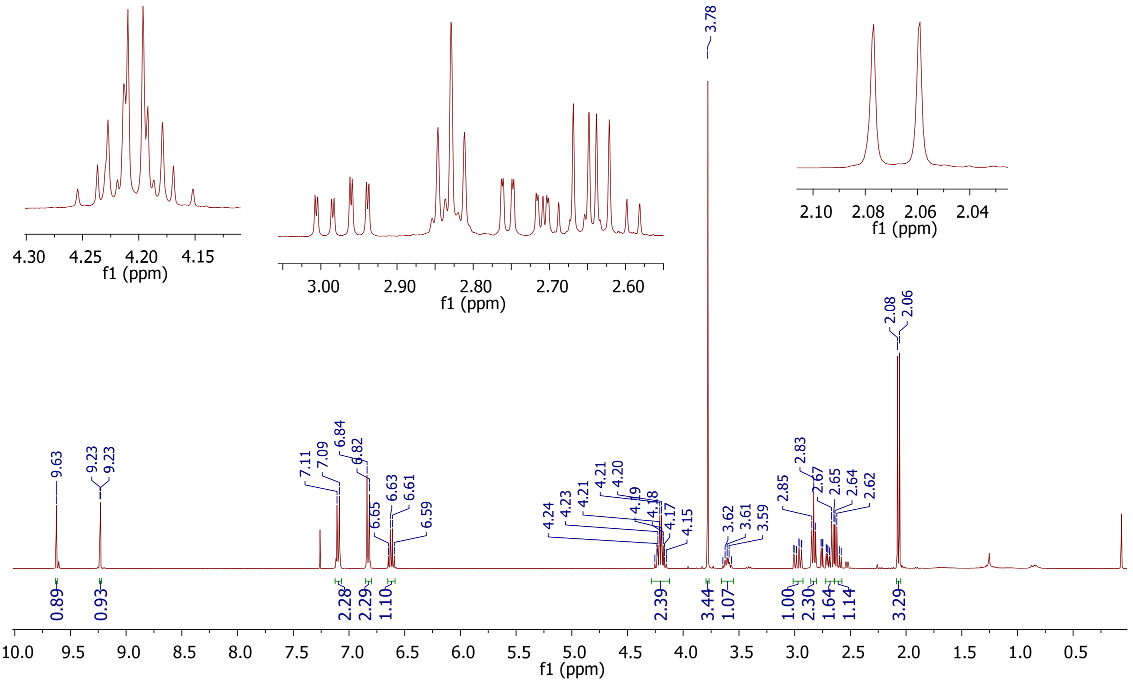

Figure S3. ^{1}H NMR of (–)-methyl-oleocanthal (**Met-OLE**) in deuterated chloroform.

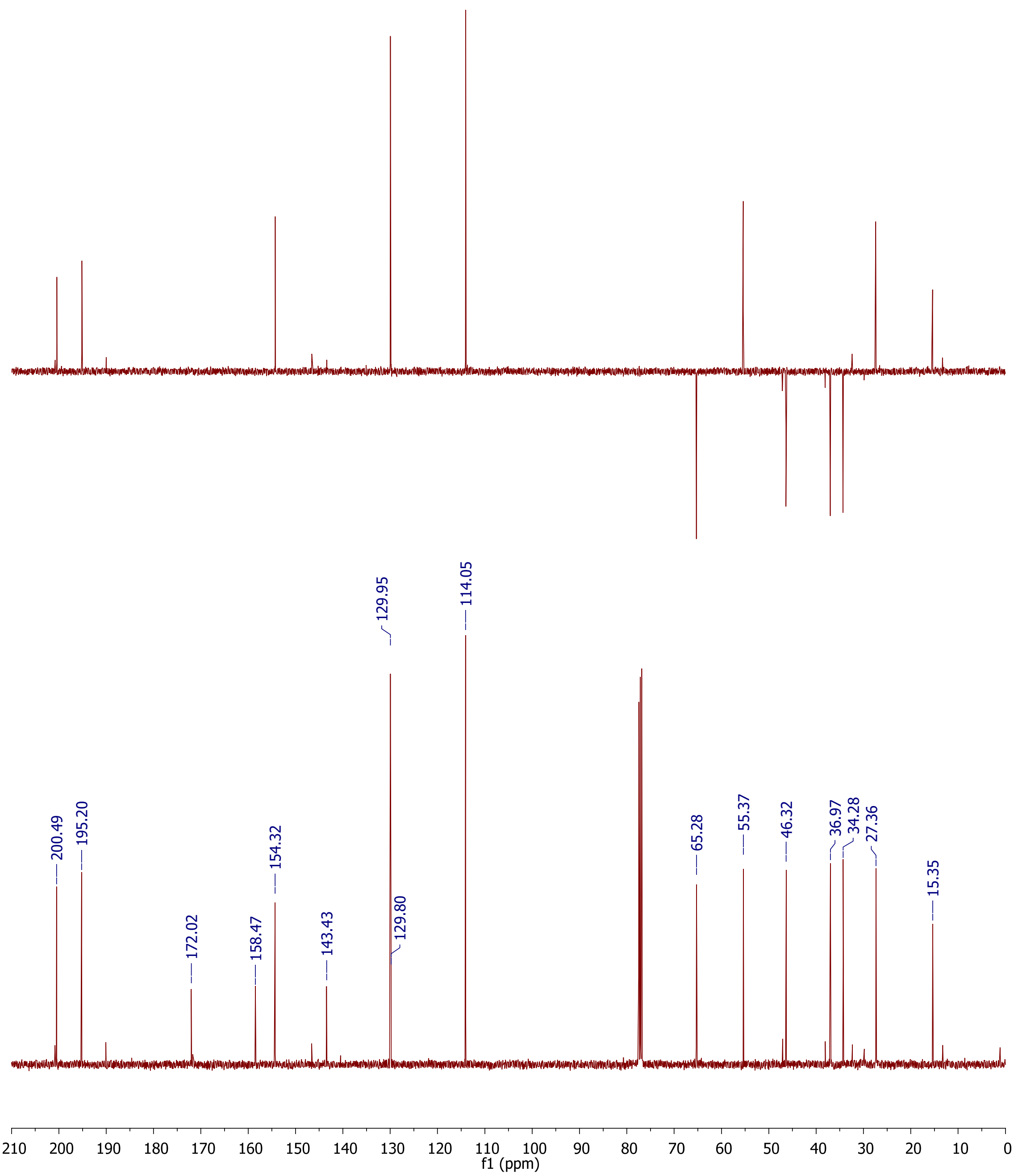

Figure S4. ^{13}C NMR (bottom) and Dept-135 (top) of (–)-methyl-oleocanthal (**Met-OLE**) in deuterated chloroform.

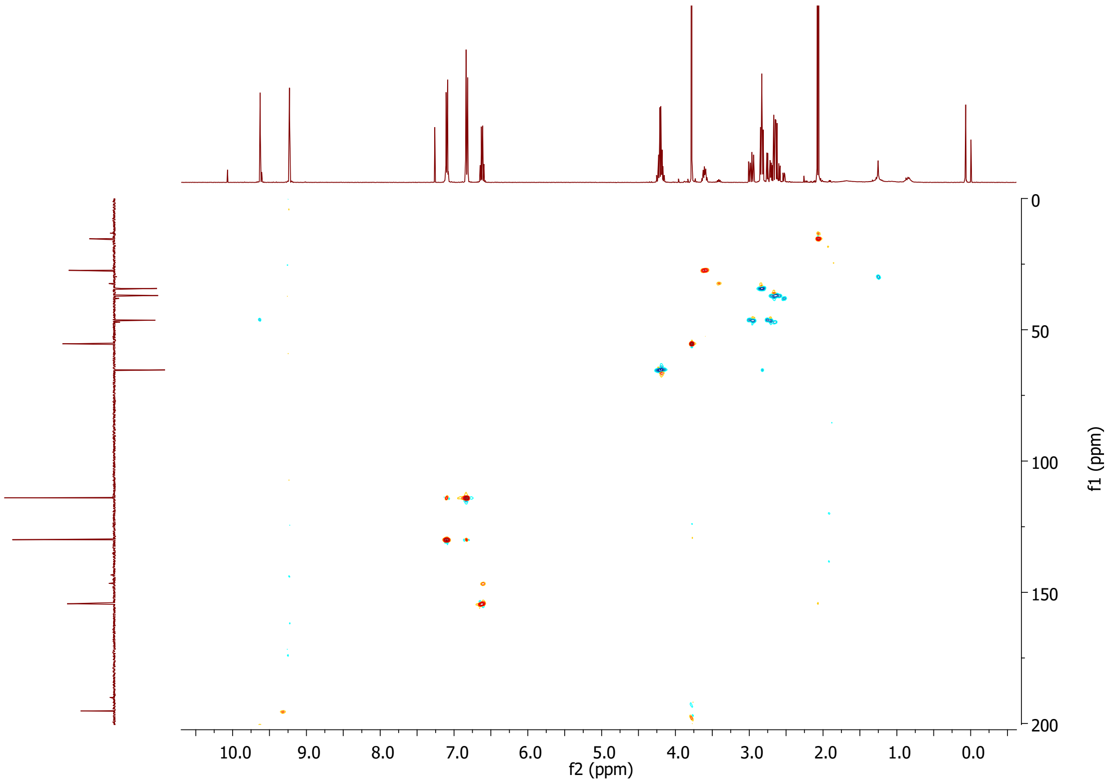

Figure S5. ^{1}H-Dept-135 (HSQC) of (–)-methyl-oleocanthal (**Met-OLE**) in deuterated chloroform.

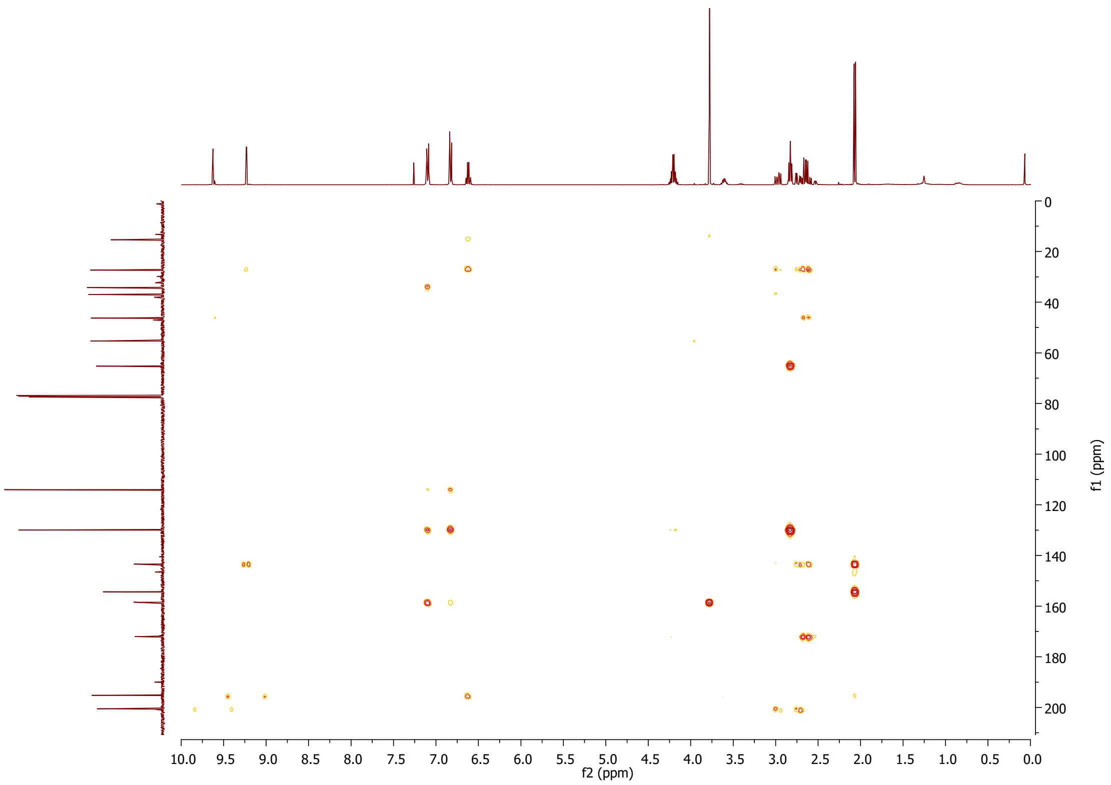

Figure S6. ^{1}H-^{13}C NMR (HMBC) of (–)-methyl-oleocanthal (**Met-OLE**) in deuterated chloroform.

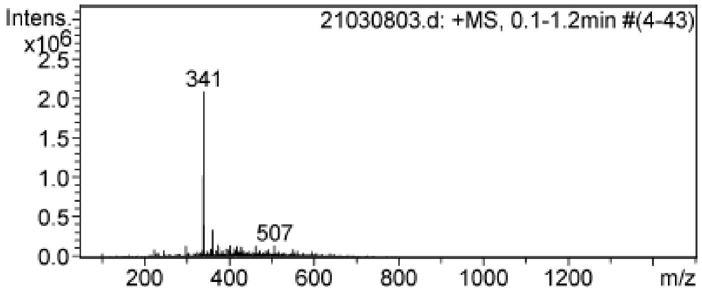

Figure S7. ESIMS of (–)-methyl-oleocanthal (**Met-OLE**).

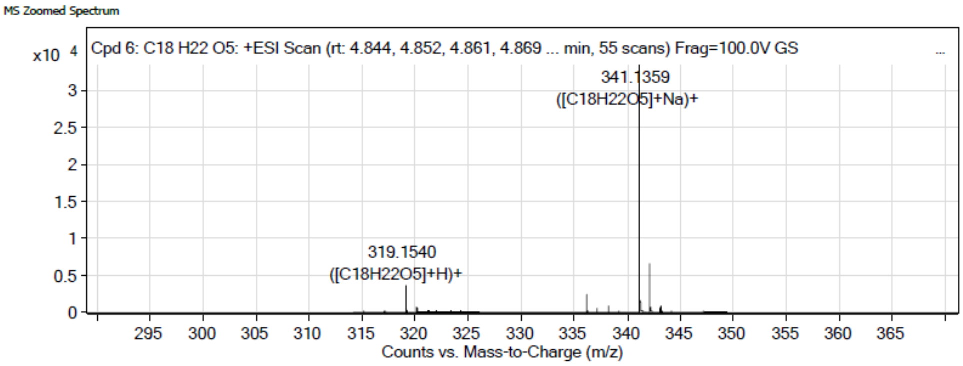

Figure S8. HRMS of (–)-methyl-oleocanthal (**Met-OLE**).

(–)-Methyl-ligustroside

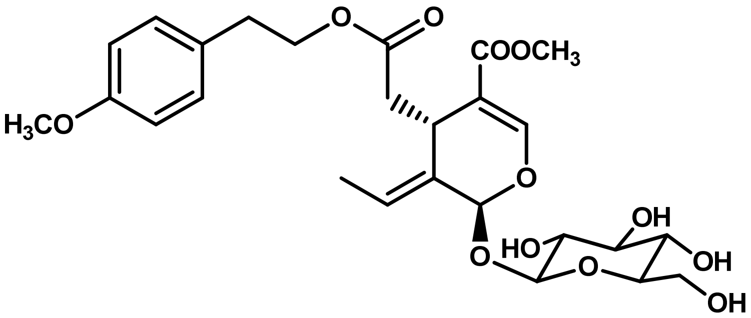

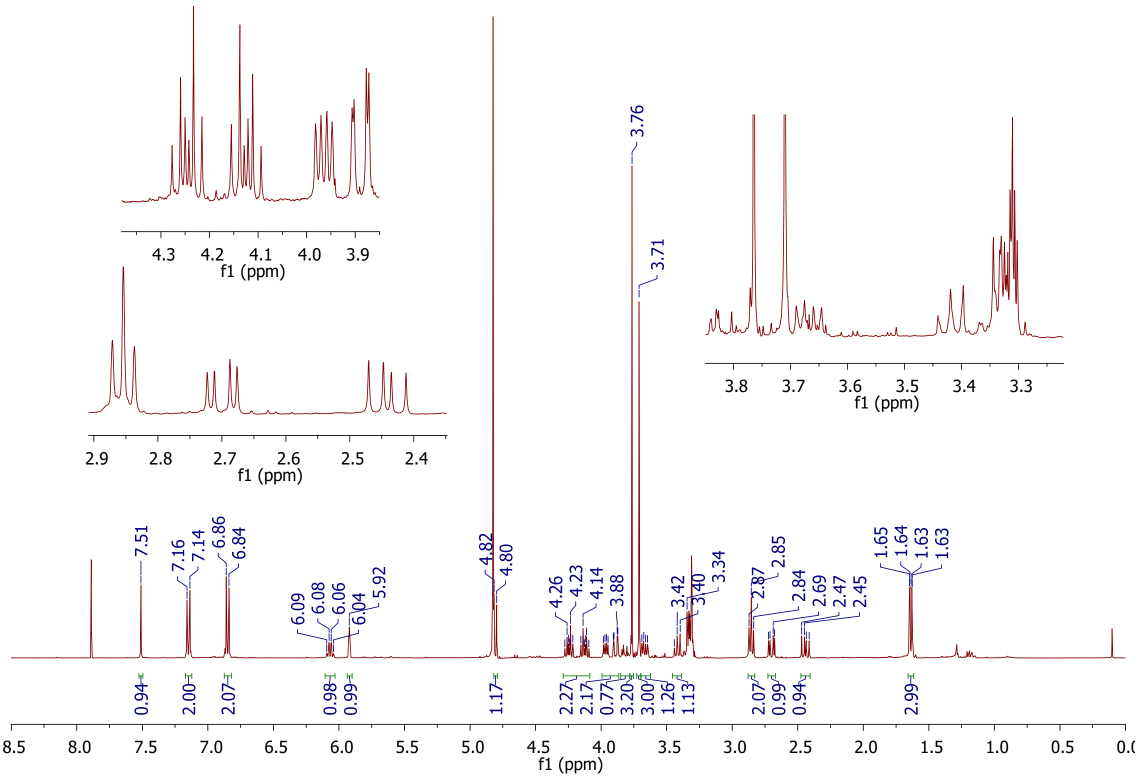

Figure S9. ^{1}H NMR of (–)-methyl-ligustroside in deuterated chloroform.

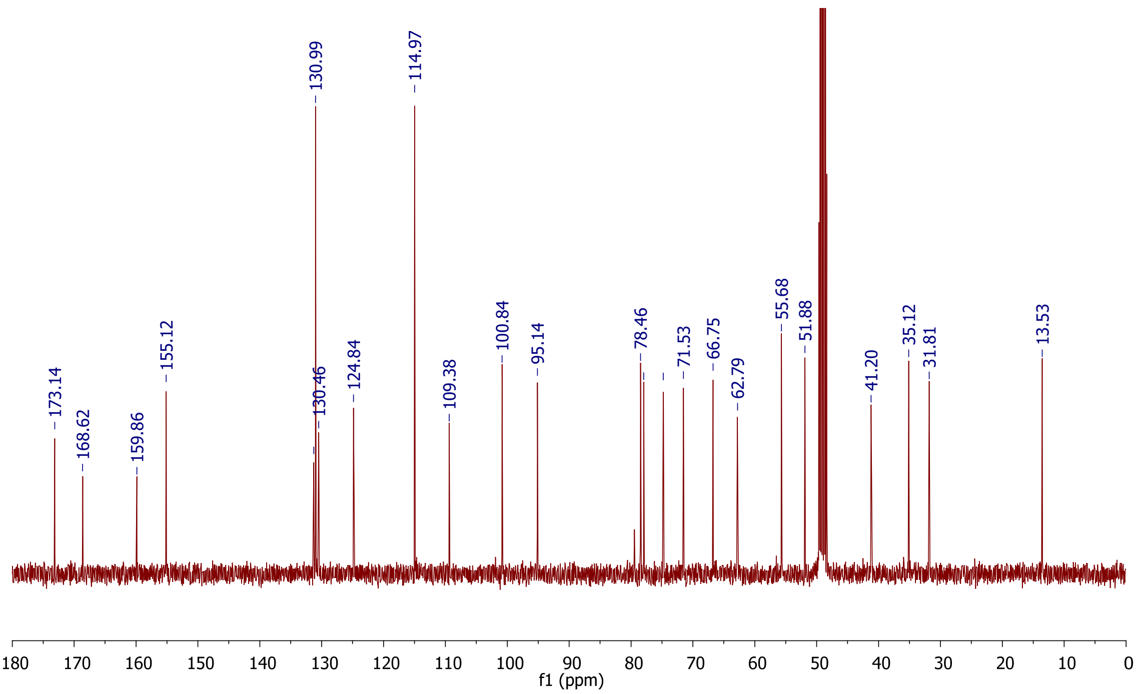

Figure S10. ^{13}C NMR of (–)-methyl-ligustroside in deuterated chloroform.

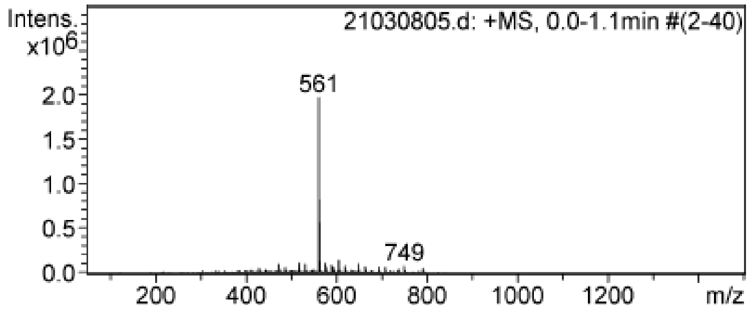

Figure S11. ESIMS of (–)-methyl-ligustroside.
